# Supplementary material for: Hepatitis E Virus in Domestic Ruminants and Virus Excretion in Milk—A Potential Source of Zoonotic HEV Infection
Source: Viruses. 2024 Apr 26;16(5):684. doi: 10.3390/v16050684 (PMC11126035; doi:10.3390/v16050684)
Supplement: Supplementary file 1 [file viruses-16-00684-s001.zip › viruses-2960298-supplementary.pdf]

Table S1. Seroprevalence of Hepatitis E virus in ruminants

Country	Seroprevalence rate (%)			Ig & method of detection	Reference
	Cattle	Sheep	Goat		
**Brazil**	1.42%	0%	0%	IgG (in-house)	[67]
**Bulgaria**	7.7%	32.2%	24.4%	total Ig (Wantai)	[60]
**Burkina Faso**	26.4%	12.0%;	28.4%	total Ig (MP Bio)	[76]
	5.1%	-	-	total Ig (MP Bio)	[77]
**China**	6.5%	-	7.5 %	total Ig (Wantai)	[69]
	18.7%	12.4%	-	total Ig (Wantai)	[70]
	28.2%	-	10.4%	total Ig (Wantai)	[71]
	29.35%	9.8%		total Ig (Wantai)	[72]
	-	35.20 %	-	total Ig (Wantai)	[52]
	25.29%<br>14.87%	9.30%	-	total Ig (Wantai)	[85]
	-	28.98%	-	n/a	[86]
	-	-	46.7%	IgG (Peking Dingguo)	[59]
	-	-	3.57% /14.29%	2 in-house assays: IgM/ IgG	[62]
	6%	-	24%	IgG (in-house)	[88]
	6.3%	-	0%	total Ig (in-house)	[92]
**Egypt**	21.6%	4.4%	9.4%	IgG (in-house)	[75]
**India**		100%/78%	100%/100%	2 in-house assays: IgG/IgG	[89]
	6.9%	-	0%	IgG (in-house)	[68]
**Italy**	-	21.3%	-	total Ig (Wantai)	[81]
	-	21.6%;	11.4%	total Ig (Wantai)	[82]
**Jordan**	14.5%	12.7%	8.3%	total Ig (Fortress Diagnostics)	[74]
**Laos**	6.8%;	-	5%(1/20)	total Ig (MP Bio)	[115]
**Nigeria**	0%	10.5%;	37.2%;	IgG and IgM (Diagnostic Automation)	[79]
	-	31.8%		IgG (ID Vet)	[87]
	0%	-	0%	IgG (ID Vet)	[80]
**Portugal**	-	16.6%	-	total Ig (Wantai)	[83]
**South Korea**	0%	-	-	IgG (in-house)	[78]
**Spain**	0%	1.92%	0.6%;	IgG (in-house)	[65]
	-	2.1%	13.8%	total Ig (MP Bio)	[84]
**Turkey**	16.5%	5.0%		IgG (SinoGeneClon)	[73]
**USA**	20.4%	-	-	Total Ig (Wantai)	[66]
	-	-	16%	IgG (in-house)	[90]
